# Generating and capitalizing on the demographic dividend potential in sub-Saharan Africa: a conceptual framework from a systematic literature review

**DOI:** 10.12688/gatesopenres.13176.1

**Published:** 2020-09-25

**Authors:** Carolina Cardona, Jean Christophe Rusatira, Xiaomeng Cheng, Claire Silberg, Ian Salas, Qingfeng Li, David Bishai, Jose G. Rimon

**Affiliations:** 1 Department of Population, Family and Reproductive Health, Johns Hopkins Bloomberg School of Public Health, 615 N. Wolfe Street, Baltimore, MD, 21205, USA; 2 Bill and Melinda Gates Institute for Population and Reproductive Health, Johns Hopkins Bloomberg School of Public Health, 615 N. Wolfe Street, Baltimore, MD, 21205, USA; 3 Department of International Health, Johns Hopkins Bloomberg School of Public Health, 615 N. Wolfe Street, Baltimore, MD, 21205, USA

**Keywords:** demographic dividend, demographic transition, fertility decline, economic growth, sub-Saharan Africa

## Abstract

**Background: **Africa will double its population by 2050 and more than half will be below age 25. The continent has a unique opportunity to boost its socioeconomic welfare. This systematic literature review aims to develop a conceptual framework that identifies policies and programs that have provided a favorable environment for generating and harnessing a demographic dividend. This framework can facilitate sub-Saharan African countries’ understanding of needed actions to accelerate their demographic transition and capitalize on their demographic dividend potential.

**Methods: **The search strategy was structured around three concepts: economic development, fertility, and sub-Saharan Africa. Databases used included PubMed and EconLit. An inductive approach was employed to expand the reference base further. Data were extracted using literature records following a checklist of items to include when reporting a systematic review suggested in the Preferred Reporting Items for Systematic Reviews and Meta-Analyses (PRISMA) Statement.

**Results: **The final review consisted of 78 peer-reviewed articles, ten reports from the gray literature, and one book. Data were categorized according to relevant demographic dividend typology: pre-dividend and early-dividend. The results from the literature review were synthesized into a framework consisting of five sectors for pre-dividend countries, namely 1) Governance and Economic Institutions, 2) Family Planning, 3) Maternal and Child Health, 4) Education, and 5) Women's Empowerment. An additional sector, 6) Labor Market, is added for early-dividend countries. These sectors must work together to attain a demographic dividend.

**Conclusions: **A country's demographic transition stage must guide policy and programs. Most sub-Saharan African countries have prioritized job creation and employment for youth, yet their efforts to secure a productive labor market require preliminary and complementary investments in governance, family planning, maternal and child health, education, and women’s empowerment. Creating a favorable policy environment for generating and capitalizing on a demographic dividend can support their stated goals for development.

## Introduction

Population projections estimate that Africa will double its current population by 2050, and that the population below age 25 will account for more than half ( WPP, 2020). Manpower is perhaps Africa’s greatest and most crucial resource to bolster the economy and stimulate development; however, the continent needs to empower the youth and harness this manpower by developing the right socioeconomic and political environments to take advantage of this resource ( African Union Commission, 2017b).

The term ‘demographic dividend’ was coined by David Bloom when analyzing the economic boom of East Asian countries between 1965 and 1990. Bloom identified that a persistent decline in fertility rates, followed by changes in the population age structure, were a common factor among Asian countries. As such, the demographic dividend is the result of changes in the population age structure that occur from declines in both child mortality and fertility during the third stage of the demographic transition period. This stage is characterized by a rapidly growing population with declining birth rates and low death rates, which increases the number of working-age individuals compared to the number of youth dependents ( Notestein, 1983). This large working-age cohort has the potential to boost the economy and improve living standards if a favorable policy environment is established. Thus, a demographic dividend presents countries the opportunity to accelerate economic growth and achieve sustainable development and social change.

The majority of nations in sub-Saharan Africa (SSA) (34 out of 48), representing nearly 75% of the continent's population in 2015, have already embarked on a population transition and are not expected to transition from one demographic stage to another before 2050 ( WPP, 2020). The first stage of the demographic transition is characterized by high fertility and infant mortality rates. In the second stage, mortality decreases, which causes a decline in fertility in the third stage. Both fertility and mortality are low in the fourth stage ( Reher, 2011). In SSA, fertility levels began to decline in the mid-1970s from an average of 6.8 children per woman to 4.8 children by the mid-2010s ( WPP, 2020). Most nations will continue to experience a decline in fertility and an increase in the working-age population over the coming decades. These countries have the unique opportunity to grow their economies and capitalize on the benefits of the demographic dividend as a result of changes in population dynamics.

In order to fully harness the benefits produced by the demographic transition, countries require targeted investments in human, social, and physical capital ( Bloom & Finlay, 2009; Bloom & Williamson, 1998; Bloom *et al.,* 2007). Evidence has shown that countries need to approach the demographic dividend as an interrelated system in which multiple sectors work together to create a favorable policy environment.

There have been multiple efforts to develop a demographic dividend framework to identify the areas that make a favorable policy environment. The African Union developed a roadmap that identifies labor, education, health, governance, and youth empowerment as fundamental pillars of the demographic dividend ( African Union Commission, 2017a). Population Reference Bureau (PRB) developed a toolkit framework to improve the understanding of the demographic dividend ( PRB, 2017). Health, education, economics, and governance were the four necessary areas identified by PRB to realize the dividend for sub-Saharan African countries. A more recent assessment by the World Bank identified evidence-based policies and programs that can catalyze the demographic transition in the Sahel region ( Shekar *et al.,* 2016). The World Bank review categorized policies and programs into three-time horizons. In the short-term, countries should catalyze the fertility transition; the medium-term period should focus on ensuring that girls are educated, and that women are empowered; and the long-term period is characterized by job creation and greater attention to pensions and savings.

Similar efforts have been placed on developing forecasting tools to quantify the potential economic gains from a demographic dividend. DemDiv allows policymakers in high-fertility countries to model different scenarios of combined investments in family planning, education, and the economy to harness the potential benefits of the demographic dividend ( HPP & USAID, 2014). This tool was developed by the Health Policy Project (HPP). LINKAGE is a multi-sectoral, multi-country and multi-agent dynamic recursive Computable General Equilibrium (CGE) model developed by the World Bank ( van der Mensbrugghe, 2011). This model was not specifically developed for the demographic dividend, but it is well suited for it ( Ahmed & Cruz, 2016).

These efforts have allowed the policy community to focus on designing strategies that can harness the benefits of a demographic dividend. Despite the evidence-based utility of these strategies and tools, to-date, there have been no systematic reviews to identify the elements that constitute a favorable policy environment to generate and harness a demographic dividend for African countries. Moreover, the suggested policies and programs are not differentiated by demographic transition stage. This systematic review aims to establish a conceptual framework to identify and critically appraise a set of policies and programs that have allowed high fertility countries to generate and attain a demographic dividend. This framework can facilitate countries in SSA to accelerate their demographic transition and capitalize on their countries’ demographic dividend potential.

## Methods

A systematic literature review was used to identify relevant scientific research and gray literature that identify a set of components that may enable a conducive policy environment to cultivate and harness the benefits of a demographic dividend. Our search strategy was based on an iterative approach consisting of both inductive and deductive methods. We developed search terms that were applied to two databases for the deductive approach. For the inductive approach, we identified a set of landmark papers to trace the studies that were most cited. Ethical approval was not required for this study. All articles are publicly available, and no primary data was gathered nor generated in this study.

### Search strategy

Using a deductive search approach, peer-reviewed articles which assess the components that enable a favorable policy environment to harness the benefits of a demographic dividend were identified from PubMed and EconLit. Table 1 presents the search strategy employed for PubMed; a similar approach was followed for EconLit. The search was structured around three concepts, Economic Development, Fertility, and Sub-Saharan Africa. For the replicable search, we used the terms "economic growth" or "economic development" with the following combinations: "high fertility", "fertility reduction", "fertility decline", "demographic dividend", and "Sub-Saharan Africa".

**Table 1.** Search strategy implemented on PubMed on 09/26/2018 to assess the relationship between fertility and economic development.

Database	Concept	Key Words		Results	Results limited to English and 10-years
PubMed	Economic Development	Line 1	(demographic dividend[Title/Abstract] OR demographic transition[Title/Abstract] OR economic growth[Title/Abstract] OR economic development[Title/Abstract])	371	34
	Fertility	Line 2	(high fertility[Title/Abstract] OR fertility reduction[Title/Abstract] OR smaller family [Title/Abstract] OR fertility decline[Title/Abstract])		
	Sub-Saharan Africa	Line 3	(sub Saharan Africa[Title/Abstract] OR Nigeria[Title/Abstract] OR Kenya[Title/Abstract] OR Senegal[Title/Abstract] OR Ethiopia[Title/Abstract] OR Rwanda[Title/Abstract] OR Tanzania[Title/Abstract])		

The literature search returned 371 articles from PubMed and 351 articles from EconLit. Searches were conducted on 09/26/2018 on PubMed and on 10/01/2019 on EconLit. The search was then restricted to only include literature published in English in the last ten years, without any country restrictions. All articles were screened independently based on title and abstract examination. Disagreements in relevancy of content were solved jointly. Given that the search on EconLit was performed a year after the PubMed search, we conducted a second screening on EconLit articles based on the relevance for early and pre-dividend countries and on its "new" contribution of the articles not seen in PubMed. Table A-1 (see *Extended data*; Cardona *et al.*, 2020) shows the list of articles that were found relevant in the first screening process, but later removed in the second screening process due to content overlap with PubMed search.

Following the identification of research articles from PubMed and EconLit, an inductive approach consisting of a "snowball search" was employed to expand the reference base further. Reference lists were reviewed to identify a set of landmark papers in the demographic dividend literature that were not listed in neither the PubMed nor EconLit results. This list is shown in Table A-2 (see *Extended data*). Additionally, reports and articles from gray literature sources such as the World Bank, the United Nations Population Fund (UNFPA), and the African Union, were downloaded and reviewed. The World Bank report on "Development Goals in an Era of Demographic Change" was the basis for all World Bank research included in this review ( Ahmed & Cruz, 2016; Ahmed *et al.,* 2014; Ahmed *et al.,* 2016; Cruz & Ahmed, 2018; Shekar *et al.*, 2016).

### Data extraction

Data were extracted using literature records following the checklist of items to include when reporting a systematic review, as suggested in the Preferred Reporting Items for Systematic Reviews and Meta-Analyses (PRISMA) Statement ( Moher *et al.,* 2009). The items included in the records were title, abstract, introduction (rationale and objectives), methods (information sources, data collection process, data items, analytical approach), results, remarks and future research, and relevance to policy.

Records were developed independently by each reviewer. Extracted data were color-coded into three categories—when possible. The three categories were: i) measurement; ii) policy interventions; and iii) key messages. Tables A-3 to A-6 (see *Extended data*; Cardona *et al.*, 2020) synthesize the literature records, they report objectives of the study, location, methodology, and key findings. To address potential reviewer bias, the summaries presented in the *Extended data* ( Cardona *et al.*, 2020) were conducted by a reviewer that did not participate in the data extraction process. These summaries were developed in parallel while the other reviewers were developing the framework.

Data from the literature records were used to develop the DD framework. We analyzed the color-coded categories to identify key concepts and trends between them to inform final conclusions on the main sectors and their components of the framework. For example, concepts such as fertility, contraception, contraceptive methods, family planning, pregnancy postponement, were classified into the family planning sector. The key sectors and interventions that we included for inclusion were based on its relevance to the demographic transition and its linkage to socio-economic growth and people’s welfare. The final sectors identified in the framework were jointly decided by both the data extraction team and the independent reviewer that developed the summaries presented in the *Extended data* ( Cardona *et al.*, 2020).

## Results

### Search results

**Figure 1.**
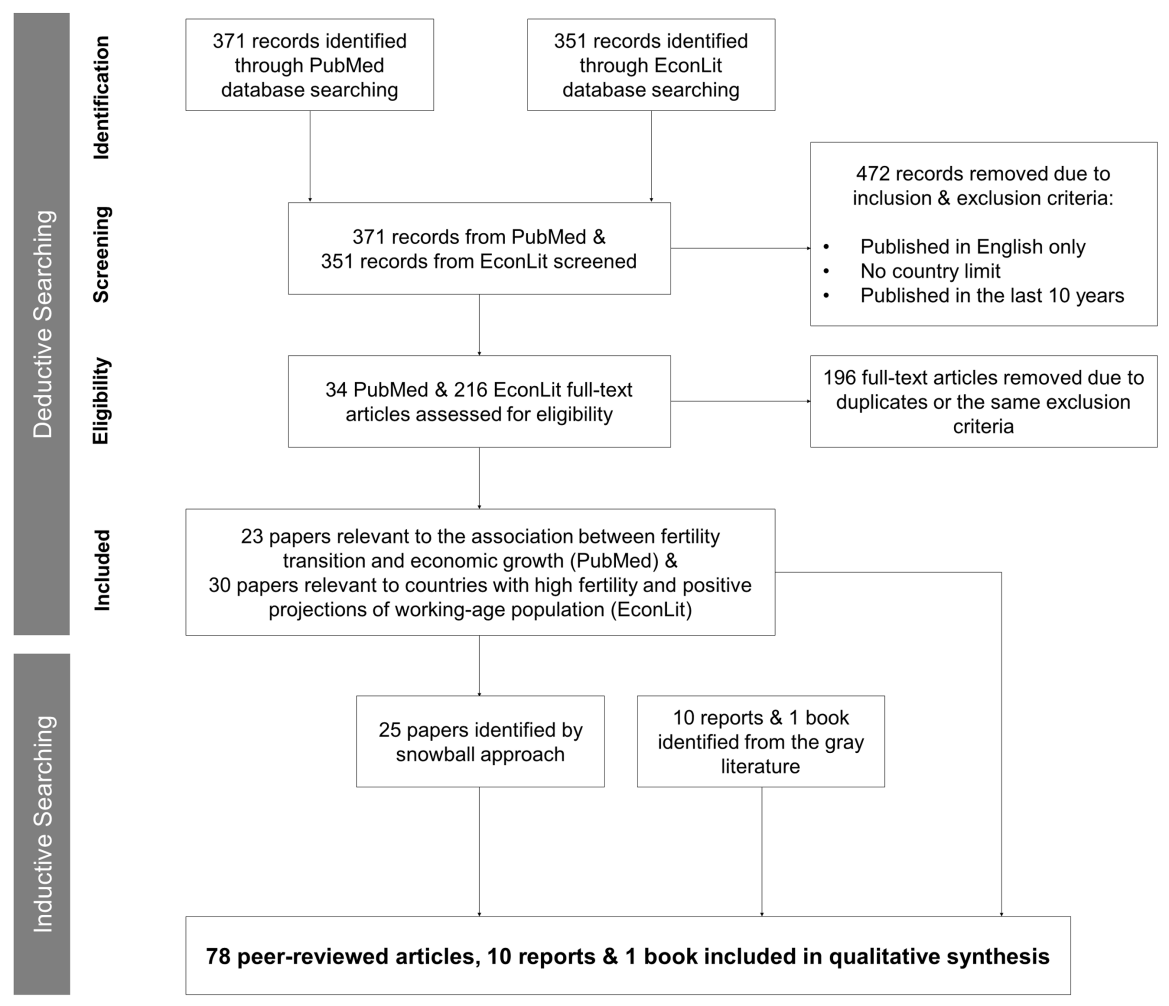
Flow of information through the different phases of the systematic review.

The PRISMA Statement guided the reporting of results from the systematic review ( Moher *et al.*, 2009). Figure 1 presents the flow of information through the different phases of the review. The initial search resulted in 371 articles from PubMed and 351 articles from EconLit. Language and time restrictions narrowed the search to 34 articles on PubMed and 216 articles on EconLit. In the eligibility phase, 23 papers from PubMed were found relevant to the association between fertility transition and socioeconomic growth and were included in the review. Of the 216 articles screened from EconLit, 46 papers were considered relevant to the demographic dividend literature in the first screening. In the second screening, 15 articles were removed, resulting in a sample of 30 articles relevant for countries with high fertility and positive projections of the working-age population. The inductive approach resulted in the identification of 25 articles. The final review consisted of 78 peer-reviewed articles, ten reports from the gray literature, and one book.

### Synthesis of results

We developed a framework that identifies the main sectors needed to create a favorable policy environment to harness the benefits of a demographic dividend. The World Bank identifies four demographic dividend typologies based on demographic characteristics, similar to the demographic transition, and future development potential ( Ahmed *et al.*, 2016). Low- and lower-middle-income countries with high fertility are characterized as pre- and early-dividend countries, while upper-middle and high-income countries make the late- and post-dividend typology. All sub-Saharan African countries are either in the pre-dividend or early-dividend typology ( WBG, 2016). A country is classified to be in a pre-dividend typology when the working-age population is projected to grow within the next 15 years, and when the total fertility rate is above four. Early-dividend countries follow a similar definition, except they have a total fertility rate below four.

**Figure 2.**
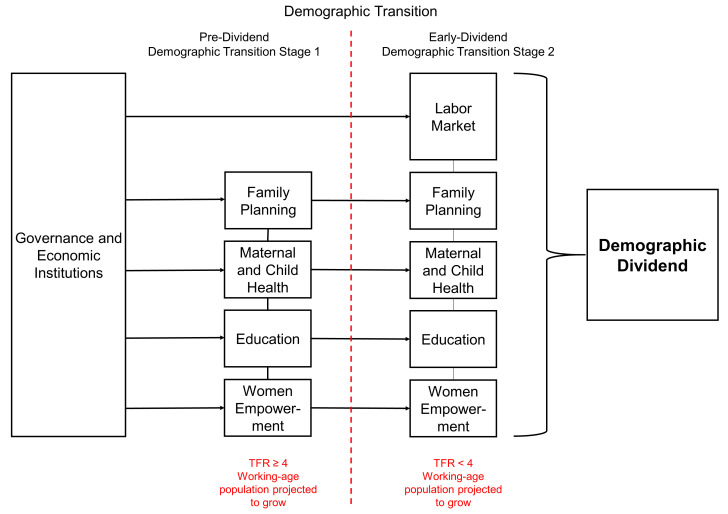
A demographic dividend framework.

**Figure 3.**
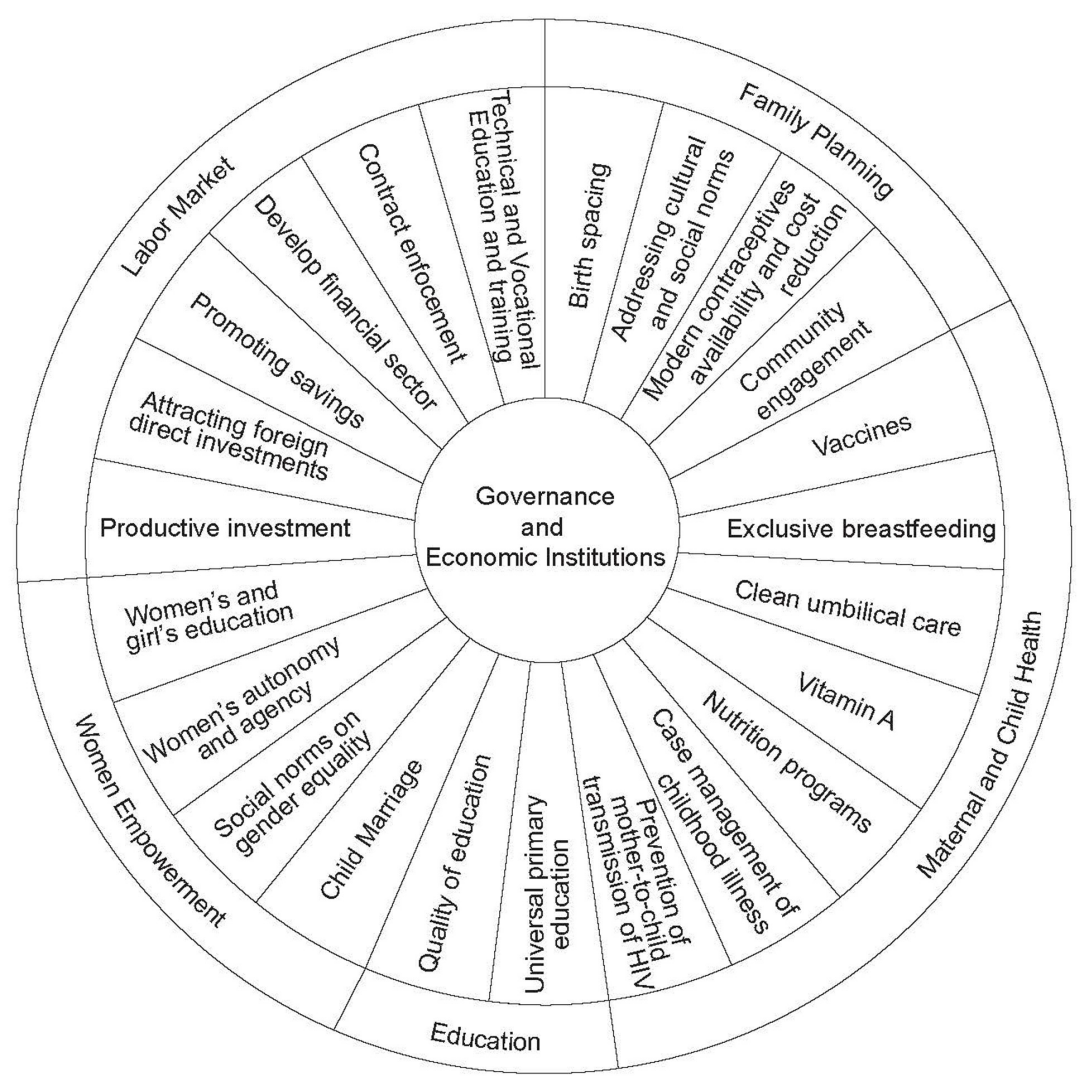
A demographic dividend framework, wheel of prosperity that summarizes potential sector-specific programs and interventions.

The framework is organized into two stages, based on the demographic dividend typology, see Figure 2. The horizontal axis of the framework represents the stages of how countries transition from high fertility to lower fertility levels. Governance and Economic Institutions is a core sector for all countries, regardless of their demographic dividend typology, which is why it is located at the far left and is the origin of the arrows that go into the other sectors. The first stage highlights key strategic sectors that pre-dividend countries should prioritize to harness the benefits of a demographic dividend. These sectors are Family Planning, Maternal and Child Health, Education, and Women's Empowerment. In addition to these four sectors, the second stage of the framework adds the Labor Market. During the early-dividend countries, the labor market is needed to capture the large ‘youth bulge’ in the population pyramid. The labor market creates the potential to increase productivity levels among youth entering the labor market. The wheel of prosperity, Figure 3, showcases a set of sector-specific programs, interventions, and areas for investment, to catalyze and harness a demographic dividend.

### Governance and economic institutions

Regardless of whether a country is classified as pre-or early-dividend, much of the literature agrees that countries can harness greater gains from a demographic dividend if certain preconditions are in place ( African Union Commission, 2017b; Bloom *et al.,* 2014; Canning *et al.,* 2015; Shekar *et al.*, 2016; UNFPA, 2019). Because these are preconditions, they are a central piece to harnessing a demographic dividend, and thus located as a cross cutting sector in the framework. Changes in the population age structure are not guaranteed to produce economic growth; a favorable policy environment is required. Bloom & Canning (2008) highlight the case of Latin America, a region that missed the opportunity to exploit its demographic window of opportunity due to high inflation, political instability, adversarial labor relations, and an inward orientation with respect to trade.

Good governance and strong institutions can encourage civil participation and enable policies that will make a favorable policy environment across all economic sectors to benefit from the demographic dividend ( Bloom *et al.*, 2014; Shekar *et al.*, 2016; UNFPA, 2019). Political stability and the rule of law are part of good governance practices which help create a climate to attract foreign direct investments that will be catalyzed to job creation and development. Institutions have the capacity to support (or block) the development of policies to realize the growth potential created by the transition ( Guengant & May, 2013), and institutional quality is correlated with growth ( Bloom *et al.,* 1999).

Financial institutions can promote a culture of savings and implement contractual savings to finance the education of children and youth ( NASEM, 2016; Ssewamala, 2015). In turn, savings can be transformed into domestic investments that will fuel growth and development, or serve as a cushion to overcome the lack or limited availability of safety nets during periods of unemployment and old age ( Canning *et al.*, 2015; Guengant & May, 2013). Economic stability can also increase the desire to postpone having children as the opportunity cost of raising children increases ( NASEM, 2016). In Nicaragua, a poverty-reduction program reduced the hazard of a birth by 15%, with a higher impact in high parity women ( Todd *et al.,* 2012).

The multi-sectoral complexity of the demographic dividend poses additional challenges to close the gap between policy design and policy implementation. Political commitment and strong leadership are needed to ensure coordination across all sectors and levels. Strengthening and developing monitoring and evaluation plans for program implementation is also required to hold institutions, programs, policies, and governments accountable ( Shekar *et al.*, 2016; UNFPA, 2019). The African Union highlights the importance of improving and strengthening the civil and political space to ensure people's participation ( African Union Commission, 2017b). AU's report titled “Harnessing the Demographic Dividend through Investments in Youth” (2017b) acknowledges that "Africa needs to invest in ensuring participatory, representative and inclusive political processes as well as responsive state institutions… ensuring protection and respect for fundamental civil, political and socioeconomic rights of young people including young women".

Bloom & colleagues (2014) also highlight the importance of a carefully constructed trade policy. Evidence from East Asian countries shows that relatively open and competitive markets contributed to East Asia's financial success, while in Latin America, a closed economic policy limited poverty reduction efforts ( Bloom *et al.,* 2009; Canning *et al.*, 2015).

### Family planning

Family planning programs are highly effective at managing fertility levels to allow couples to attain their desired family size. In addition, they have the potential to improve social conditions in countries with historically high fertility levels ( Bloom *et al.*, 2010; Eastwood & Lipton, 2011; Li *et al.,* 2018). Among these social improvements, women can be more empowered to make their own decisions in the household and take ownership of their reproductive health. Focusing on fertility management, particularly in pre-dividend countries (countries with TFR ≥ 4), can accelerate a shifting age structure and pave the path to harness greater gains from a demographic dividend ( Ahmed *et al.*, 2016; Bloom *et al.*, 1999). In East Asia, around one-third of the economic growth between 1965 and 1990 was attributable to a rapid demographic transition triggered by the implementation of family planning programs ( Bloom *et al.*, 1999; Bloom *et al.*, 2014; Bloom & Canning, 2008; Bloom & Finlay, 2009; Bloom & Williamson, 1998). Population simulations for Nigeria project that a change in fertility from the UN medium-variant to the low-variant level results in an increase in income per capita of 5.6% in 20 years, and 11.9% in 50 years ( Ashraf *et al.,* 2013).

Higher socioeconomic status (SES) is positively associated with declining fertility. As families become smaller, household members have greater resource allocation per capita, which, for children, can be translated into higher educational investments and higher parental time per child ( Ahmed *et al.*, 2016; Becker & Murphy, 1988). There is a vast literature base on the relationship between high socioeconomic status and small family size; evidence comes from both developed and developing economies ( Komura, 2013; Majumder & Ram, 2015; May & Turbat, 2017; NASEM, 2016). Fertility reductions in Matlab, Bangladesh were linked to positive effects on health, earnings, and household assets of women and children ( Joshi & Schultz, 2007). In Colombia, Profamilia was able to reduce fertility rates among young aged women by between 11–12%, which resulted in an increase of 0.08 years in education attainment ( Miller, 2009). In a cross-country analysis, Lee & Mason (2010) estimated that the relationship between fertility and educational investments per child relative to income have an elasticity of -1; this means that a 10% fertility reduction can increase schooling per child by 10%. This notion has been partly attributed to the concept of quantity-quality trade-off in children ( Becker & Lewis, 1973; Behrman, 2015; Bloom & Canning, 2008; Eltigani, 2009; Majumder & Ram, 2015; NASEM, 2016; Sinding, 2009). The quantity-quality trade-off reflects a change in the behavior of the parental dyad as a result of the modernization of society. As societies transition, the cost of raising a child becomes increasingly more expensive. Therefore, parents prefer to have fewer children because they want to allocate more resources per child ( Ashraf *et al.*, 2013; Diebolt & Perrin, 2013; Galor, 2012; Herzer *et al.,* 2012; Notestein, 1983; Prettner *et al.,* 2013).

Women perceive the benefits of a smaller family size across the wealth distribution differently; these perceptions are associated with fertility trends. Evidence from Bangladesh, India, Indonesia, Nepal, Philippines, and Vietnam has found that women from lower SES had a time lag in attaining their preferred fertility compared to women from higher SES ( Majumder & Ram, 2015). This gap is partially addressed by contraceptive pricing schemes, which aim to reduce costs to benefit low-income groups, but the inequity in access and use remains high across the wealth distribution ( Campbell *et al.,* 2013; Canning *et al.*, 2015; Lewis, 1986). Government emphasis on the benefits of small family size may accelerate the time lag across income strata. This idea has already been integrated into the national plans of a few developing nations, such as Indonesia and Iran ( McKelvey *et al.,* 2012; Shekar *et al.*, 2016).

The pace of fertility decline is subject to contraceptive practices and women's demand for contraception; however, overall demand is greatly affected by the supply of quality services and methods ( Fabic *et al.*, 2015; Lewis, 1986; Mwaikambo *et al.,* 2011; NASEM, 2016). Research has found that inconsistent contraceptive availability impacts women’s contraceptive uptake ( Bongaarts & Watkins, 1996; Gertler & Molyneaux, 1994). Indonesia's demographic transition highlights the importance of modern contraceptives for achieving fertility decline. Between 1982 and 1987, contraceptive use was the dominant factor that influenced fertility decline and, thus, its demographic transition ( Gertler & Molyneaux, 1994). Therefore, ensuring the affordability and availability of contraceptives is paramount to stimulating fertility decline and attaining desired fertility.

Social norms and cultural practices are important determinants for women’s reproductive outcomes and are important considerations for successfully achieving a demographic transition. Despite declines in recent years, polygamy is still a common practice in SSA ( Shekar *et al.*, 2016). Women in polygamous relationships often time bear more children than women in monogamous relationships due to the idea that they are competing for their husband's resources ( NASEM, 2016). In many African societies, men tend to dominate decisions related to women's reproduction and contraception. Studies have shown that women are likely to obtain contraceptive methods in discreet ways, particularly unmarried women, increasing the covert use of contraceptives ( Campbell *et al.*, 2013; Shekar *et al.*, 2016). Another barrier to reducing fertility is the tradition and encouragement of large families. ( Shekar *et al.*, 2016). In communities in the Democratic Republic of Congo, it is common to have a large number of children for their own and kin's security ( Romaniuk, 2011).

Understanding the social context of a community and the local perceptions of family planning can help guide the development of successful family planning programs to reduce national fertility rates. Lessons learned from other countries to accelerate the demographic transition highlight the need for effective programming. This can be achieved by working with and training local community health workers, midwives, and community change agents ( Bloom *et al.*, 2014; Mwaikambo *et al.*, 2011; Shekar *et al.*, 2016). Evidence from high fertility regions has shown the power that social networks have in influencing fertility change through the exchange of information among individuals, or more recently, through social media. The Navrongo experiment in Ghana showed how integrating family planning at the community level was a key component to program success ( Bloom *et al.*, 2014). In Kenya, Behrman *et al.* (2002) found that social networks can shape family planning attitudes and reproductive health behaviors. They found that the odds of using contraception increased by more than two if a woman had at least one family planning user in her network. In Ethiopia, Rwanda, Kenya, Nigeria, Tanzania, Senegal, India, Bangladesh, and Indonesia, community level task-shifting involving local community health workers, midwives, and community change agents have had positive effects on family planning ( Shekar *et al.*, 2016).

Holistic family planning programs include both the provision of a range of contraceptive methods as well as comprehensive strategies to address cultural norms and beliefs related to reproductive health norms. Successful family planning programs combine initiatives in both the demand and supply side to ensure uninterrupted and equitable access to services ( May & Turbat, 2017; NASEM, 2016; Shekar *et al.*, 2016). Governments play a key role in changing the general public's attitude towards family size and in ensuring the attainment of desired fertility by guaranteeing the availability of family planning information and services to all women of reproductive age.

### Maternal and child health

Declines in infant and child mortality have a direct effect on fertility levels ( Eastwood & Lipton, 2011; Galor, 2012; Reher, 2011). Although the causal path can be argued, fertility decline could have preceded the decline in infant and child mortality ( Chowdhury, 1988; Galor, 2005). Some multi-country analyses suggest that causality runs from infant or child mortality to fertility, identifying that fertility decline ranges from 0.5 to 0.9 as a result of a fall of 50 in the infant and child mortality rate ( Conley *et al.,* 2007; Eastwood & Lipton, 2011; Lorentzen *et al.,* 2008). Reducing infant and child mortality, along with maternal mortality, is a global goal that is part of the Sustainable Development Goals and included in the national plans and policies of developing countries.

Although there is disagreement in the direction of the causal path, there is a consensus that the achievement of reduced child mortality, fertility decline, and lower maternal mortality are key to achieving fertility management and attaining a demographic dividend. Effective and equitable access to family planning affects desired fertility, children's survival, and maternal health. Access to contraception is estimated to have averted 32% of maternal deaths in 2008 in regions that have low contraceptive use ( Ahmed *et al.,* 2012). Fertility reduction is also associated with a reduction in maternal deaths. Maternal mortality and parity have a J-shape relationship in developing countries ( Jain, 2011). The risk of mortality is higher during the first birth, mainly because the majority of births occur at younger ages. The risk decreases with preceding births and above the fourth birth, the risk increases again. The J-shape relationship is also associated with what is known as "too early, too late, or too frequent" ( Ahmed *et al.*, 2012; Jain, 2011), in which women who give birth at short spacing intervals and those with multiple children are at increased risk of death. Empirical evidence has shown that eliminating births among high-risk groups could eliminate between 20% to 25% of all maternal deaths ( Winikoff & Sullivan, 1987).

Traditionally, families view children as essential labor resources that help with economic activities, such as agriculture, and as a form of security in old age ( Notestein, 1983). In countries with high child mortality, parents will desire to have more children to ensure protection against possible losses or to replace those losses ( Bloom & Canning, 2008; Conley *et al.*, 2007; Majumder & Ram, 2015; Shekar *et al.*, 2016). Fertility decline is one of the response mechanisms of declines in infant and child mortality. As societies transition into the modern world, child mortality is likely to decrease, and parents are likely to desire to have fewer children due to rising costs of raising a child ( Eastwood & Lipton, 2011).

There are multiple evidence-based interventions that can reduce infant, child, and maternal mortality. We summarize here some of the most compelling and relevant programming we found on our literature search.

Safe motherhood initiatives, vaccinations, child nutrition programs (including vitamin A), and case management of childhood illness in improving maternal and child health have been evidenced by several researchers and are considered as key interventions by governments ( African Union Commission, 2017a; Bongaarts & Casterline, 2013; Bongaarts & Watkins, 1996; Canning *et al.*, 2015; Majumder & Ram, 2015; Shekar *et al.*, 2016). In 1987, the Safe Motherhood Initiative was launched as a global campaign with the goal of reducing maternal mortality through interventions focused on making childbirth safer ( Ahmed *et al.*, 2012; Starrs, 1997). Some of the most recognized interventions include deliveries with a skilled attendant at birth, improvements in the availability and quality of comprehensive emergency obstetric care, and expanded services for women during pregnancy, childbirth, and the immediate postnatal periods. In particular, those living with HIV to prevent mother-to-child transmission ( Canning *et al.*, 2015; Wilson, 2015).

Compared to other regions, breastfeeding has been associated with longer birth intervals in Africa, and is associated with positive health gains for both mother and child ( Bongaarts & Casterline, 2013). Lactation increases the duration of postpartum amenorrhea, and long-term breastfeeding is consistently associated with long periods of postpartum amenorrhea, ovarian inactivity, and reduced fertility ( Majumder & Ram, 2015; Mwaikambo *et al.*, 2011; NASEM, 2016). Postpartum infecundability is one of the close determinants of fertility and is a result of breastfeeding and postpartum abstinence ( Bongaarts, 2015). During the period of pre-transitional fertility, couples often do not consciously practice birth control to limit their children. Natural fertility is highest in populations with a low age at marriage and short durations of breastfeeding, which are largely determined by community customs ( Bongaarts & Watkins, 1996).

Vaccines are one of the most cost-effective health technologies to improve child survival ( Bloom *et al.*, 2014; Bloom & Canning, 2008; Shekar *et al.*, 2016). In SSA, vaccines have prevented a significant number of child deaths given that infectious diseases, such as malaria, are common ( Bloom *et al.*, 2014; Eastwood & Lipton, 2011; Spence, 2009). Strengthening the community-based distribution of health technologies, such as vaccines, via existing health systems has been successful in the past and can accelerate the reduction of child deaths ( Shekar *et al.*, 2016).

Under-five mortality reduction has a direct impact on increasing life expectancy at birth. The increase in life expectancy reflects the immense efforts in reducing mortality during the first five years of life ( Bloom & Canning, 2008; Reher, 2011). The overall increase in life expectancy at birth is a reflection of public health and medical interventions, such as access to clean water and sanitation and vaccines and antibiotics. Mortality decline has been faster on average across developing countries than in historical Europe; however, the world still has a set of 11 countries with a life expectancy at birth below the age of 51, six of which are located in Africa ( Cardona & Bishai, 2018).

### Education

Countries between the second and third stages of the demographic transition have the unique opportunity to boost their economies by taking advantage of the large "youth bulge" in their populations. Investing in education and tailoring education to the labor market can increase output per work, maximize productivity, and drive development

The African Union identifies education as one of the four pillars that underpin the roadmap to harnessing the demographic dividend in Africa ( African Union Commission, 2017a). Priorities identified by the union include improving access to quality education that matches labor market demands and embracing technical education to allow individuals to harness their natural abilities and create opportunities for self-employment ( African Union Commission, 2017b).

Forecasting simulations to 2030 found that in SSA, improvements in educational attainment can increase GDP per capita by 22.4% and reduce poverty by 38.9 percentage points, compared to the baseline scenario ( Ahmed *et al.*, 2014). Cruz & Ahmed (2018) estimated that between 1950-2010, an increase of one percentage point in the working-age population boosted the GDP per capita growth rates by 1.1 to 2.0 percentage points, on average. Similar estimates were found by Bloom & Williamson (1998), with increases in GDP ranging between 1.4 and 2.0 percentage points. If the output per worker increases, the country's economy may grow remarkably ( Ahmed & Cruz, 2016; Eastwood & Lipton, 2011).

Individuals with higher levels of education are more efficient producers of health, which in turn is translated into a healthier population with higher productivity levels ( Grossman, 1972). Increased school enrollment in Malawi, Uganda, Ethiopia, and other African countries has had important implications for changing fertility rates, partly through changing desired fertility ( Behrman, 2015). Gertler & colleagues (1994) found that high levels of education in the 1970s in Indonesia, along with higher and equitable wages for both men and women, were significantly associated with reductions in the birth hazard. Women with an educational level beyond secondary school have access to higher wages, which increases the opportunity-cost between investing time in raising a child or spending time at work ( Behrman, 2015; Eastwood & Lipton, 2011; Galindev, 2011; Majumder & Ram, 2015).

Countries must work to ensure girls have access to education and are encouraged to stay in school. Novel approaches have been implemented across the region. The Kenyan Government changed the requirements of primary schooling from seven to eight years, which resulted in greater educational attainment among girls and reduced marital education gaps ( Shekar *et al.*, 2016). The implementation of universal primary education in Nigeria resulted in a reduction of 0.26 births resulting from every additional year of schooling ( Osili & Long, 2008). Tanzania is now offering free primary and secondary education to all students in the country and is committed to investing more in the education sector ( African Union Commission, 2017b).

Furthermore, the quality of education has been shown to increase economic gains ( African Union Commission, 2017b; Shekar *et al.*, 2016). According to the World Bank, one of the preconditions to catalyze a demographic dividend in Africa is to invest in the quality of education from primary, secondary, and tertiary level institutions ( Shekar *et al.*, 2016). However, one of the challenges in tracking progress on education quality is the limited availability of information, not only in Africa, but amongst all developing economies.

### Women empowerment

Creating and capitalizing on the benefits of a demographic dividend requires empowering women and girls through improvements in health, education, and decision-making power ( Canning *et al.*, 2015). Women who are empowered have the potential to transform countries' economies from a state of high fertility, low education, and sluggish economic growth towards a state of high education and fast economic growth ( Bloom *et al.*, 2009; Canning *et al.*, 2015; Prettner & Strulik, 2017). For example, one-third of the economic growth experienced by the Asian Tigers between the 1960s and 1990s has been attributed to improvements in women's empowerment and participation in the labor force as a result of a rapid fertility transition ( Bloom *et al.*, 1999; Bloom *et al.*, 2014; Bloom & Canning, 2008; Bloom & Finlay, 2009; Bloom & Williamson, 1998).

In traditional societies, married women are expected to bear children to fulfill their caregiver role and to ensure the reproduction of kin ( Folbre, 2001). Patriarchal societies tend to have a high fertility rate, while societies in which women are empowered tend to have a lower fertility rate ( Komura, 2013). Women with fewer childbearing responsibilities have more time to invest in the labor market and increase labor supply, which in turn increases household resources and welfare. This relationship is also inverse. Women who hold stable jobs and earn a high income are more likely to have fewer children because income losses during childbearing are higher for them than for women with low wages ( Ahmed & Cruz, 2016; Bloom *et al.*, 2009; Canning *et al.*, 2015; Eastwood & Lipton, 2011; Feyrer *et al.,* 2008; Herzer *et al.*, 2012).

Child marriage impedes on the rights, health, and wellbeing of young girls ( Chari *et al.,* 2017). Child marriage results in higher rates of school drop-out, less decision-making power in sexual relationships, and higher fertility rates as a result of higher exposure to marriage ( Campbell *et al.*, 2013; Canning *et al.*, 2015; Shekar *et al.*, 2016; UNFPA, 2017). Underemployment and unemployment is high among girls who marry early, resulting in an inability to earn wages and a cycle of poverty in future generations ( UNFPA, 2017). Increasing the median age at first marriage does not only reduce the exposure to pregnancy but also has the potential to increase the proportion of children who spend time in a two-parent family while growing up, which is beneficial for the long-term health of the child ( Cherlin, 1999). In Bangladesh, India, Indonesia, Nepal, Philippines, and Vietnam, the use of contraceptive methods and a higher median age of first marriage were the main drivers of fertility decline ( Majumder & Ram, 2015).

Family planning and education programs implemented at the community level have been shown to successfully delay the age of marriage in young girls; findings come from Ethiopia, Burkina Faso, and Yemen ( Engebretsen & Kaboré, 2011; Erulkar & Muthengi, 2009; Freij, 2010). These programs have shown that laws alone are not enough to delay marriage and prevent child marriage. For example, Indonesia experienced no change in the age of marriage after the National Marriage Act was passed in 1974 ( Cammack *et al.,* 1996). Programs that tackle complex cultural and economic norms within the community are important for long-term, sustainable change.

Empowered women can be characterized by various attributes such as autonomy to make their own reproductive health decisions, economic self-reliance, and household decision making ( African Union Commission, 2017b). In addition to policies and initiatives to empower women, empowering women to the full extent requires a societal shift in gender norms. To protect women's autonomy, the African Union (2017b) recommends that countries should "adopt and enforce target[s] to end all forms of discrimination and violence against all women and girls, including domestic and sexual violence as well as harmful practices such as child and forced marriage and Female Genital Mutilation"; "strengthen domestic criminal accountability, responsiveness to victims and judicial capacity"; and "affirm the primacy of international and regional human rights law and constitutional laws over religious, customary and indigenous laws as a means to ensuring women's emancipation and autonomy."

Finally, women's and girls' education are a key component of women's empowerment and an important factor in the ongoing process of fertility transition ( Shapiro, 2012). Girls’ education does not only have implications on fertility outcomes - it also directly affects employability and age at marriage ( Majumder & Ram, 2015; Shapiro, 2012). Most theories predict that better-educated women desire fewer children than less-educated women. Other arguments for the impact of women's education on fertility outcomes is the role that income plays on families' economic prospects and the improved social status that comes with higher education. Promoting women's education also improves the welfare of both parents and children by increasing the transfer of knowledge between generations and further increasing the value of the next generation's human capital ( Kim, 2016).

### Labor market

Africa has a young population; according to UN projections, more than half of the population on the continent will be below the age of 25 by 2050 ( WPP, 2020). Thus, countries need productive and supportive labor markets to optimize their production capacity to absorb the growing young population ( Shekar *et al.*, 2016). In addition, with declining fertility stimulating the demographic dividend, a "youth bulge" will be produced as a result of a large proportion of working youth compared to the number of dependent children ( Canning *et al.*, 2015; Reher, 2011; Ssewamala, 2015). Particularly, for early dividend countries (countries with TFR < 4), the youth bulge can maximize the benefits from a demographic dividend through employment ( WBG, 2016). However, when the labor market is not able to satisfy the needs of this working-age population, the youth bulge can become a burden on society, and young adults are often forced into performing work within the informal sector ( Shekar *et al.*, 2016; Turner, 2009). Similarly, the youth bulge can be pushed into the informal sector when they are undereducated and low-skilled, thereby entering the labor market unprepared ( Ssewamala, 2015).

Countries that are in the early-dividend typology, those with a total fertility rate above four children per woman and the share of the working-age population expected to grow, should focus their efforts on implementing policies and programs that benefit the labor market. However, this does not mean they have to stop investing in the five sectors prioritized in the pre-dividend era.

Projections show that in order to keep employment rates constant, the region needs to create 1.6 million jobs every month by 2030, and more than 2.0 million per month by 2050 ( NASEM, 2016). In 2015, the International Labor Organization estimated that 6 out of 100 people living in SSA were without work but available for and seeking employment. Countries should be able to create jobs at a rate that exceeds the rate of population growth in order to achieve full employment ( Ahmed *et al.*, 2016).

There is a dearth of jobs within formal labor markets in low-resource settings, and jobs for youth and women are scarce ( Earl *et al.,* 2017). Formal labor markets have to support the population through laws that protect both employees and employers ( Guengant & May, 2013). However, countries need to consider the risks and benefits of their labor policies. Bloom & colleagues (2003) found that rules governing the hiring and firing of workers can limit employers from taking risks; minimum wage above the market price can discourage hiring and training; and labor-management bargaining can trigger labor market inertia.

The benefits from a demographic dividend can be greater if young adults are able to accumulate human capital for when they enter the labor force and if the labor market is able to offer productive jobs ( African Union Commission, 2017b; Ahmed *et al.*, 2016; Bloom *et al.*, 2014; UNFPA, 2017). Nigeria is an interesting case study that experienced a mismatch between skill sets and labor demand. Unemployment rates were higher among young adults with secondary education than among young adults with primary education ( Reed & Mberu, 2014). However, Nigeria's economy has been slowly shifting from concentrating its labor force in agricultural activities to the services sector, which is associated with a more developed economy ( WBG, 2010). This shift has also occurred throughout SSA but at a slower pace. In the early 1990s, workers in the agricultural sector accounted for 63% of the labor force, which decreased to 55% by 2015, while workers in the services sector increased by six percentage points. Countries in SSA need to focus on building skill sets that match the demands of the labor market, which includes technical and vocational education and training ( African Union Commission, 2017a; Ahmed *et al.*, 2016; Shekar *et al.*, 2016; UNFPA, 2017). For example, Tanzania is currently implementing "The Education and Skills for Productive Jobs (ESPJ)" program to close the mismatch between labor demands, labor supply, and skills shortages in the job market ( African Union Commission, 2017b).

As mentioned in the previous section, women's participation in the labor market is crucial for greater gains in the economy, and it has a multiplier effect on human capital and population health. Countries must strive to create a favorable policy environment and provide incentives for female workers to find employment in the formal sector ( Bloom *et al.*, 2010). Throughout the 1990s and 2000s, the participation of women in the labor market has been a great contributor in maximizing the benefits from a demographic dividend across different developing countries ( Ssewamala, 2015).

Participation in the formal labor market provides benefits beyond economic growth and development. Workers that are not part of the formal labor market are unlikely to have contributed to a retirement fund. When this population of informal workers reaches an old age and are no longer in conditions to work, they need to rely on their personal savings to live ( Dramani & Oga, 2017; Shekar *et al.*, 2016). However, if they were unable to save money during working-age, they have to rely on family members or on the state– if the state has a welfare system in place.

When fertility levels are low, a low youth-dependency ratio can be harnessed through higher savings rates that have the capacity to boost a faster physical accumulation ( Ashraf *et al.*, 2013; Eastwood & Lipton, 2011; Guengant & May, 2013; Turner, 2009). Not being able to save or have a pension becomes an incentive to have more children in which parents can rely during old age ( Jiang *et al.,* 2013). Most countries in SSA present an unsustainable mix of poor saving rates and low capital productivity ( Eastwood & Lipton, 2011). The Asian Tigers' experience has shown that changes in the labor force per capita, in the savings rate, and in the investment rate are three mechanisms through which a changing age structure may affect the rate of economic growth ( Bloom & Williamson, 1998). It is estimated that in African countries with high fertility, a one percentage point increase in the working-age population share has the capacity to increase the savings share of GDP by 0.8 percentage points while reducing poverty by 0.76 percentage points ( Ahmed *et al.*, 2016).

Financial markets that provide financial education and incentives to encourage household savings are important ( Ssewamala, 2015; Turner, 2009). Novel approaches to encourage savings with a special focus on the youth have promising results. A randomized control trial in Uganda found that adolescents that were given the opportunity to have a savings account were more likely to save for their education than those that did not receive a youth savings account ( Curley *et al.,* 2010). In addition, participants became encouraged about their futures. Other examples from developing countries are Equity Bank in Kenya, Barclays Bank in Ghana, Khan Bank and Zoos Bank in Mongolia, and Banco Nacional de Bolivia in Bolivia ( Ssewamala, 2015). Most of these programs restrict withdrawal until reaching age 18 and provide incentives to save. Some of the incentives include double interest if no withdrawal in a quarter, free banker's checks to pay school fees, and matched savings with varying match rates.

The fourth stage of the demographic transition is characterized by low birth and death rates in which population growth is virtually zero or even negative ( Reher, 2011). During this period, there is no longer a youth bulge that can push the economy. In the third transitional stage, the living standards of the population tends to increase, and individuals are more likely to live longer. In return, people are more likely to increase their savings to ensure monetary security in the future ( Bloom & Canning, 2008). Both in India and China, longer lifespans have been associated with increased savings for retirement that were accompanied by favorable trade policies and favorable investment rates ( Bloom *et al.*, 2010). The economic mechanisms by which people in different age groups produce, consume and save resources are of crucial importance for the demographic dividend to occur and for societies to accumulate generational wealth. Central to demographic dividend and generational economy are work, consumption, sharing and saving ( Lee & Mason, 2011).

## Discussion

The majority of SSA countries are in the first or second stage of their demographic transition, which positions them as pre- and early-dividend countries ( Ahmed *et al.*, 2016; WBG, 2016). Informed by the results of this review, a framework was created to guide policy and interventions for SSA countries to capitalize on their demographic dividend potential. For countries in the pre-dividend typology, this framework proposes five essential sectors for investment and action, namely 1) Governance and Economic Institutions, 2) Family Planning, 3) Maternal and Child Health, 4) Education, and 5) Women's Empowerment. An additional sector, 6) Labor Market, is added for countries in the early dividend typology. Given the limited resources that have to be distributed across sectors, the framework proposes a set of potential priority policy levers and intervention areas summarized in the wheel of prosperity.

The timing of policy and interventions is essential for countries to reap the demographic dividend. Good governance and strengthening economic institutions provide countries with the opportunity to exploit the potential of their demographic dividend through civic participation, abiding by the rule of law, and attracting foreign investment to create jobs and boost the economy ( Bloom *et al.*, 2010). Failure to avoid high inflation and political stability shadows the opportunities to take advantage of the demographic dividend potential ( Bloom & Canning, 2008). In the majority of countries that are in the pre-dividend typology, income per capita is low, along with low contraceptive uptake, and high rates of illiteracy and child marriage with slow or no progress ( Ahmed *et al.*, 2016; Goodkind *et al.,* 2018; Guengant & May, 2013; Li & Rimon, 2018). Such countries can realize their demographic dividend if proper policies and interventions are simultaneously implemented to ensure that women and families are able to make informed decisions on when and how many children to have and attain desired fertility levels. Early-dividend countries should focus on the labor market, but also explore strategies and practices to accelerate the reduction of maternal and infant mortality, ensure universal access to quality education and provide optimal support for women empowerment and, in particular, eliminate child marriage ( Behrman, 2015; Campbell *et al.*, 2013; Kim, 2016; Osili & Long, 2008; Prettner *et al.*, 2013; Shekar *et al.*, 2016; UNFPA, 2017).

Ensuring continued progress is a priority to harness the benefits of a demographic dividend. Countries in the early dividend typology, such as Rwanda and Ghana, have managed to stabilize their economies, establish stronger institutions across sectors with remarkable effort to tackle corruption, and kept positive strides in political stability, family planning, maternal and child health, education, women empowerment and gender equality ( AU, 2015; Canning *et al.*, 2015; NASEM, 2016). However, the decline in fertility and the increasing number of people entering the labor market requires intentional efforts from stakeholders to establish policies and programs to facilitate the participation of young people in the labor market. As this new generation transitions to the work environment, policymakers have to establish mechanisms to allow the new generation of workers, in particular women, to equally participate, stay in the labor market, and develop the skillset that matches labor demand ( Komura, 2013; Mberu & Reed, 2014; Prettner & Strulik, 2017; Ssewamala, 2015). Countries have to nurture their policies and interventions to ensure that the education system matches the availability of jobs in the labor market. Through vocational trainings, countries have the opportunity to capitalize on the large number of people entering the labor market by diversifying the sources of national wealth while ensuring the creation of jobs as well as providing people with the skills to self-employ ( Fox *et al.,* 2016; Kabagenyi *et al.,* 2015; UNFPA, 2017; WBG, 2016). Further, the culture of saving plays an integral part in the process of taking advantage of the demographic dividend. Regrettably, despite a few success stories from a few banks and microfinance institutions based in SSA, this culture is still deficient in the majority of SSA countries and will require transformational actions across nations ( Shekar *et al.*, 2016; Ssewamala, 2015).

Our review has identified and critically appraised existing evidence on policy and programs that have created a favorable environment to generate and harness the demographic dividend. These sectors vary by countries' demographic transition stage, and they all work in synergy for the benefits of the demographic dividend to occur. The benefits of a demographic dividend can go on for generations but can only happen from a synergistic multi-sectoral approach ( Dramani & Oga, 2017). Countries have the potential to create a strong basis for prosperous generations through a continuum of the human capital development and generational wealth from households' and countries' savings ( Bloom & Canning, 2008). The case of the Asian Tigers may not necessarily be applicable to the SSA countries. Still, it can serve as a learning opportunity of capitalizing on the demographic transition and taking timely steps to create a better socioeconomic status for the current and future generations. Countries that successfully harness their potential for a first demographic dividend in their second and third stages of the demographic transition have the potential to reap even more benefits from a second demographic dividend in their fourth stage. This potential is a result of savings during working ages, which in the end fosters capital accumulation and economic growth ( Canning *et al.*, 2015).

### Strengths and limitations

Our review has unique strengths. First, to our knowledge, this the first systematic literature review in the demographic dividend literature that stratifies findings by demographic transition stage and demographic dividend typology to establish a multi-sectoral framework. Our review also identifies sector-specific programs and interventions. Second, the combination of literature from public health, economics, and gray literature provides holistic evidence for demographic dividends benefits beyond just economic benefits. Third, a hybrid between a deductive and inductive approach for identifying literature allowed for the inclusion of key landmark papers in the demographic dividend literature from leading institutions such as the World Bank, which are not necessarily published as peer-reviewed articles.

Our study has several limitations. First, we limited our public health search to PubMed and the economic search to EconLit. These databases are the largest for public health literature and economic literature, respectively, although the content of these databases may not be exhaustive for the existing literature about demographic dividend. Second, we cannot account for studies that are not available online that might be generated by NGOs or local country offices or governments. Thirdly, the review only included literature published in English, which may leave behind local documents. Finally, the rapidly changing drivers of global economies and challenges that did not exist when the reviewed literature was produced may limit the applicability of our findings in some contexts.

## Conclusion

This review establishes a demographic dividend framework with a set of sectors that may allow countries to capitalize on their demographic dividend potential. The country's demographic transition stage is a crucial element to guide policy and action. Although the majority of countries across SSA have prioritized job creation and employment, for the countries to fully take advantage of their demographic dividend potential, a strategic multi-sectoral approach will be required. Supporting women's and children's health and intentional simultaneous development across family planning, maternal and child health, education, women empowerment, and the labor market, will be of crucial importance for SSA to reap the demographic dividend. The proposed framework is a tool to guide countries in nurturing their long-term and strategic plans, and the proposed wheel of prosperity can guide short-term and mid-term policy design and program implementation. Nevertheless, more research is needed to expand the evidence base on effective policies and interventions that would accelerate progress towards attaining the demographic dividend in SSA-specific contexts.

## Data availability

### Underlying data

All data underlying the results are available as part of the article and no additional source data are required.

### Extended data

Harvard Dataverse, Gates Institute Demographic Dividend, Supplementary files - Generating and capitalizing on the demographic dividend potential in sub-Saharan Africa: a conceptual framework from a systematic literature review, https://doi.org/10.7910/DVN/F3WL99 ( Cardona *et al.*, 2020).

This project contains the following extended data:

- Table A-1.docx (List of articles from EconLit included in the first and second screening)
- Table A-2.docx (List of papers considered as landmark in the demographic dividend literature)
- Table A-3.docx (Synthesis of literature records retrieved from PubMed)
- Table A-4.docx (Synthesis of literature records retrieved from EconLit)
- Table A-5.docx (Synthesis of literature records retrieved from Snowball Approach)
- Table A-6.docx (Synthesis of literature records retrieved from the Gray Literature)
- References.docx (References for 78 articles included in the final review)

### Reporting guidelines

Harvard Dataverse: PRISMA checklist for “Generating and capitalizing on the demographic dividend potential in sub-Saharan Africa: a conceptual framework from a systematic literature review”. https://doi.org/10.7910/DVN/F3WL99 ( Cardona *et al.*, 2020).

Data are available under the terms of the Creative Commons Zero "No rights reserved" data waiver (CC0 1.0 Public domain dedication).
