# Supplementary material for: Generating and capitalizing on the demographic dividend potential in sub-Saharan Africa: a conceptual framework from a systematic literature review
Source: Gates Open Res. 2020 Sep 25;4:145. [Version 1] doi: 10.12688/gatesopenres.13176.1 (PMC8028847; doi:10.12688/gatesopenres.13176.1)
Supplement: Peer review history

# Peer review: Reviewer response for version 1

**DOI:** 10.21956/gatesopenres.14375.r30225

**Published:** 2021-04-06

**Authors:** Eva Berde

**Affiliations:** 1 Department of Economics, Corvinus University of Budapest, Budapest, Hungary

After considering several aspects and pre-selecting 722 articles, the article analyzed papers on sub-Saharan Africa countries in terms of demographic dividend potential based on 5 + 1 criteria, considering 78 journal articles and gray publications.

 The selection of 5 monitored sectors is perfect in pre-dividend and early dividend countries: 1) Governance and Economic Institutions, 2) Family Planning, 3) Maternal and Child Health, 4) Education, and 5) Women’s Empowerment. In early dividend countries, the addition of a sixth sector, Labor Market, is fully justified. This sector would also play an important role in pre-dividend countries, I suggest that Labor Market could be included in their analysis of the pre-dividend countries as well. We know that economic policy in these countries does not yet have the strength to introduce measures that affect the labor market, but the development of the labor market itself will largely determine where intervention is needed in the future.(see citation).

 Overall, the highlighting of key aspects in the article has been very successful and shows the reader well the potential for development in the countries of sub-Saharan Africa.

 The Discussion chapter continues to cite articles, although we basically expect the authors to express their own views in the discussion. The main points of the cited articles and the aspects that authors consider important from their own approach when explaining the topic should be summarized in the Discussion part.

 Formally contradictory, but the content is true: ‘Economic stability can also increase the desire to postpone having children as the opportunity cost of raising children increases’. Because of the apparent contradiction, it would be worth justifying the truth of the claim.

 Overall, the article is an extremely valuable piece of work, I only advise making the above minor modifications.

Are the rationale for, and objectives of, the Systematic Review clearly stated?

Yes

Is the statistical analysis and its interpretation appropriate?

Not applicable

Are sufficient details of the methods and analysis provided to allow replication by others?

Yes

Are the conclusions drawn adequately supported by the results presented in the review?

Yes

Reviewer Expertise:

My research topic relevant to the article is the role of the demographic dividend in economic growth. I have several other research topics.

I confirm that I have read this submission and believe that I have an appropriate level of expertise to confirm that it is of an acceptable scientific standard, however I have significant reservations, as outlined above.

# Peer review: Reviewer response for version 1

**DOI:** 10.21956/gatesopenres.14375.r30227

**Published:** 2021-01-18

**Authors:** Adebusuyi Isaac Adeniran

**Affiliations:** 1 Department of Sociology and Anthropology, Obafemi Awolowo University, Ile-Ife, Osun State, Nigeria; 2 United Nations Office on DrugS and Crime (UNODC), Vienna, Austria

The paper is both timely and well-structured. Appropriate methodological/operationalization basis has been established for the paper; though improved primary sources of data would have made the paper more appealing. The study's results are well presented and aptly discussed.

 I recommend that the paper should be indexed as it is. Meanwhile, if there would still be space for further data collection, I would recommend that primary sources of data should be explored to further compliment the secondary sources that have been utilized robustly by the authors.

Are the rationale for, and objectives of, the Systematic Review clearly stated?

Yes

Is the statistical analysis and its interpretation appropriate?

Yes

Are sufficient details of the methods and analysis provided to allow replication by others?

Yes

Are the conclusions drawn adequately supported by the results presented in the review?

Yes

Reviewer Expertise:

migration, mobility, African studies, technology and society, regional integration, economic growth, youth development, governance, poverty, public policy and international development

I confirm that I have read this submission and believe that I have an appropriate level of expertise to confirm that it is of an acceptable scientific standard.

# Peer review: Reviewer response for version 1

**DOI:** 10.21956/gatesopenres.14375.r29688

**Published:** 2020-11-19

**Authors:** Latif Dramani

**Affiliations:** 1 CREG-CREFAT, University of Thiès, Thies, Senegal

The authors' problem is the optimization of SD in SSA in a context where the continent faces an opportunity for socio-economic growth induced by a strong young population. Therefore, the aim of their article is to develop a conceptual framework comprising the policies and programs relevant to generating and exploiting a demographic dividend.

 The subject is topical, especially for the geographical area chosen as the unit of analysis. Indeed, Sub-Saharan Africa faces the challenge of management and of taking charge of its population, which mainly consists of young people.

 Addressing how to take up this challenge while optimizing its demographic potential and providing a conceptual framework for having the latest information on the issue is an asset for researchers and development.

 The methodology used is essentially based on a literature review of mainly relevant articles published over the past ten years on the "PubMed" and "EconLit" sites using the keywords: "economic development", "fertility" and "sub-Saharan Africa". The Preferred Reporting Items for Systematic Reviews and Meta-Analyzes (PRISMA) evaluation method of Moher *et al*.(2009), which allows the reader to see the strengths and weaknesses of a research work, was selected. After consultation only 78 scientific articles met the criteria; 10 reports from the gray literature and one book.

 The authors find the results very interesting and instructive.

 Definition of two types of countries according to their situation in relation with the demographic dividend: countries with an early demographic dividend and countries with a predictive demographic dividend. This ranking is based on six key factors, and the simultaneous implementation of which is essential to achieve the demographic dividend.

 These factors are: 1) Governance and economic institutions, 2) Family planning, 3) Maternal and child health, 4) Education, 5) Women's empowerment and 6) labor market. The latter is intended for countries with early DD which differentiates it from countries with predictive SD.

**Substantive remarks:**

 From a substantive perspective, the research objective is clearly defined and the literature used appropriate to the objective of the study. It is topical and refers to key researchers in the field.

 The added value of this work lies in its nature as a handbook on demographic dividend issues, and constitutes a pioneering study.

 However, some substantive limitations should be noted:

1. The inclusion and exclusion criteria in the methodology should be more clearly defined.
2. Retaining nothing but papers published in scientific journals is an important limitation in the approach used. Indeed, the theme of the demographic dividend is relatively recent and there is not yet a critical mass of researchers at country level who can publish research in journals of a certain rank.
3. It would be interesting to include the national reports published by the countries on the theme of the demographic dividend. These official reports published by the ministerial departments of the countries allow to know the level of country commitment on the DD theme and its real inclusion in the national development plan of the countries.
4. Specify the publication dates of the documents consulted in the abstract.
5. The results found are interesting, but the recommendations resulting from this research were not clearly formulated.
6. A major limitation of this study is that the number of documents reviewed may not be sufficient to develop a framework to promote the optimization of DD in Sub-Saharan Africa.
7. Much of the evidence on capturing the demographic dividend based on country reports produced with UNFPA support in more than 24 countries in West and Central Africa using the NTA methodology was obscured from the documents consulted.
8. Some of the recent development publications, the Demographic Dividend Monitoring Index (DDMI), are completely absent from the documents consulted. Naturally this is linked to the methodology used which creates a systematic bias taking into account the criteria retained for the selection of articles. Thus, for example the Sahel countries which are in the process of leading the Sahel Women Empowerment and Demographic Dividend (SWEDD) initiative, although they have set up demographic dividend observatories with very advanced analysis tools based on the generational economy have been overlooked. As the countries most engaged on the continent in capturing SD, incorporating the experience of these countries would undoubtedly have improved understanding of the normative framework the authors propose.
9. This paper could have assessed the effectiveness of the policies and programs carried out within the framework of the optimization of DD in SSA.

Are the rationale for, and objectives of, the Systematic Review clearly stated?

Yes

Is the statistical analysis and its interpretation appropriate?

Partly

Are sufficient details of the methods and analysis provided to allow replication by others?

Partly

Are the conclusions drawn adequately supported by the results presented in the review?

Partly

Reviewer Expertise:

Generational Economy, Macroeconomy, poverty

I confirm that I have read this submission and believe that I have an appropriate level of expertise to confirm that it is of an acceptable scientific standard, however I have significant reservations, as outlined above.
